# Supplementary material for: An mRNA Vaccine for Herpes Zoster and Its Efficacy Evaluation in Naïve/Primed Murine Models
Source: Vaccines (Basel). 2025 Mar 19;13(3):327. doi: 10.3390/vaccines13030327 (PMC11946168; doi:10.3390/vaccines13030327)
Supplement: Supplementary file 1 [file vaccines-13-00327-s001.zip › vaccines-3491545-supplementary.pdf]

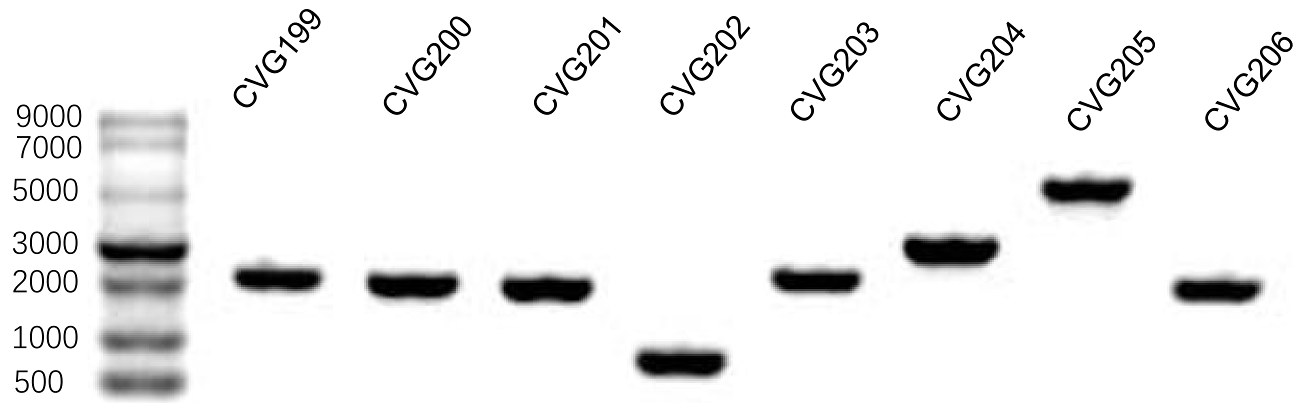

RNA electrophoresis Densitometry

	Area	Mean	Min	Max	IntDen	RawIntDen	IntDen Ratio /CVG206
**CVG199**	1020	123.02	0	255	125480	125480	0.994
**CVG200**	1020	140.629	0	255	143442	143442	1.136
**CVG201**	1020	144.106	0	255	146988	146988	1.164
**CVG202**	1020	144.169	0	255	147052	147052	1.165
**CVG203**	1020	125.048	0	255	127549	127549	1.010
**CVG204**	1122	150.365	0	255	168709	168709	1.336
**CVG205**	1122	126.598	0	255	142043	142043	1.125
**CVG206**	1122	112.548	0	255	126279	126279	1.000
